# Stool frequency recording in severe acute malnutrition (‘StoolSAM’); an agreement study comparing maternal recall versus direct observation using diapers

**DOI:** 10.1186/s12887-017-0874-0

**Published:** 2017-06-07

**Authors:** Wieger Voskuijl, Isabel Potani, Robert Bandsma, Anne Baan, Sarah White, Celine Bourdon, Marko Kerac

**Affiliations:** 1 Department of Pediatrics & Child Health, the College of Medicine, University of Malawi, Blantyre, Malawi; 2 Global Child Health Group, Emma Children’s Hospital, Academic Medical Center, Meibergdreef 9, 1105 AZ Amsterdam, The Netherlands; 3 Division of Gastroenterology, Hepatology and Nutrition, The Hospital for Sick Children, Toronto, Canada; 4 Department of Public Health, College of Medicine, University of Malawi, Blantyre, Malawi; 5 Leonard Cheshire Disability & Inclusive Development Centre, University College London, London, UK; 6 Department of Population Health, London School of Hygiene & Tropical Medicine, London, UK

**Keywords:** Stool frequency, Maternal recall, Diarrhoea, Diapers, Equivalence trial, Severe acute malnutrition, Malnutrition

## Abstract

**Background:**

Approximately 50% of the deaths of children under the age of 5 can be attributed to undernutrition, which also encompasses severe acute malnutrition (SAM). Diarrhoea is strongly associated with these deaths and is commonly diagnosed solely based on stool frequency and consistency obtained through maternal recall. This trial aims to determine whether this approach is equivalent to a ‘directly observed method’ in which a health care worker directly observed stool frequency using diapers in hospitalised children with complicated SAM.

**Methods:**

This study was conducted at ‘Moyo’ Nutritional Rehabilitation Unit, Queen Elizabeth Central Hospital, Malawi. Participants were children aged 5–59 months admitted with SAM. We compared 2 days of stool frequency data obtained with next-day maternal-recall versus a ‘gold standard’ in which a health care worker observed stool frequency every 2 h using diapers. After study completion, guardians were asked their preferred method and their level of education.

**Results:**

We found poor agreement between maternal recall and the ‘gold standard’ of directly observed diapers. The sensitivity to detect diarrhoea based on maternal recall was poor, with only 75 and 56% of diarrhoea cases identified on days 1 and 2, respectively. However, the specificity was higher with more than 80% of children correctly classified as *not* having diarrhoea. On day 1, the mean stool frequency difference between the two methods was −0.17 (SD; 1.68) with limits of agreement (of stool frequency) of −3.55 and 3.20 and, similarly on day 2, the mean difference was −0.2 (SD; 1.59) with limits of agreement of −3.38 and 2.98. These limits extend beyond the pre-specified ‘acceptable’ limits of agreement (±1.5 stool per day) and indicate that the 2 methods are non-equivalent. The higher the stool frequency, the more discrepant the two methods were. Most primary care givers strongly preferred using diapers.

**Conclusions:**

This study shows lack of agreement between the assessment of stool frequency in SAM patients using maternal recall and direct observation of diapers. When designing studies, one should consider using diapers to determining diarrhoea incidence/prevalence in SAM patients especially when accuracy is essential.

**Trial registration number:**

ISRCTN11571116 (registered 29/11/2013).

**Electronic supplementary material:**

The online version of this article (doi:10.1186/s12887-017-0874-0) contains supplementary material, which is available to authorized users.

## Background

Malnutrition in all its forms (fetal growth restriction, suboptimum breastfeeding, stunting/wasting and micronutrient deficiencies) is widespread and is directly or indirectly responsible for approximately 45% of deaths in children aged under 5 years that occur globally [1]. In low-income countries, especially those affected by HIV/AIDS and tuberculosis, mortality in children with severe acute malnutrition (SAM) remains high and tackling this is an international priority [2].

Apart from co-morbidities such as HIV or tuberculosis, diarrhoea is common among children with severe malnutrition [3–6] leading to prolonged admission and high mortality rates [6]. Recent reports from multiple countries noted a prevalence between 50 and 60% in children with SAM [3–6]. Our clinical impression is that the prevalence of diarrhoea is similar in our department, i.e., the Nutritional Rehabilitation and Research Unit (“MOYO”) at the Department of Pediatrics at Queen Elizabeth Central Hospital (QECH), Blantyre, Malawi. Diarrhoea is strongly associated with adverse clinical outcome [6] 1 and is frequently used as an outcome measure in research studies as well as in daily clinical practice. Therefore, a reliable and valid method of stool frequency assessment is of pivotal importance [7]. Currently most centres rely on maternal recall of stool frequency and consistency to assess the presence and severity of diarrhea [7] (defined as ≥3 loose or watery stools/day [8]. Based on our own observations and unpublished reports from other nutrition rehabilitation centres we postulated that the maternal recall for determining stool frequency is associated with high observer variability and significant reporting bias.

To ensure we and others in Nutritional Rehabilitation and Research Units (NRU) in low income countries are making clinical decisions on the basis of the best possible information, the aim of the current trial was to determine whether stool frequency as assessed by ‘maternal recall’ was equivalent [9] to a ‘directly observed method’ in which a health care worker directly observed stool frequency using diapers in children with SAM. Complementing this, we assessed maternal preference for stool frequency assessment method.

## Methods

### Characteristics of participants

This study was conducted at ‘Moyo’ NRU at Queen Elizabeth Central Hospital (QECH), the academic teaching hospital at the College of Medicine (COM), University of Malawi. As well as being a referral centre for the whole Southern Region of Malawi, ‘Moyo’ provides inpatient SAM treatment services for the whole of Blantyre urban and rural district, covering a population of approximately 1 million. Participants were children aged 5–59 months admitted with SAM defined as: weight-for-height ≤ −3 Z-scores (WHO growth standards) *and/or* a mid-upper-arm circumference (MUAC) of <115 mm (non-oedematous malnutrition, “marasmus”), *and/or* nutritionally induced bilateral pitting oedema (oedematous malnutrition, “kwashiorkor” and “marasmic kwashiorkor”). Oedema was defined as: Oedema level-I (+) is bilateral pitting oedema affecting the ankles/ft; level-II (++) affects both feet, hands, lower arms and lower legs; and level-III (+++) is generalized bilateral pitting oedema including both feet, legs, arms and face.

All patients had *complicated* SAM; with medical complications like systemic or respiratory infection, gastroenteritis or HIV disease. Those with uncomplicated SAM would have been treated as outpatients in community-based treatment programs in Blantyre district [10].

### Design

**Fig. 1:**
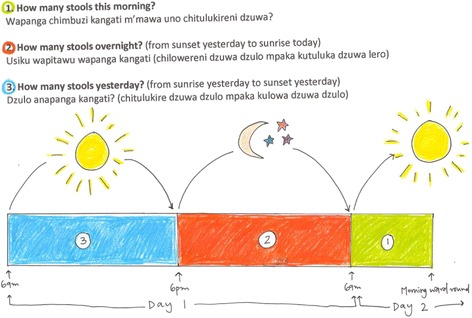
Simple picture aid used to help primary care giver recall stool frequency of child with SAM

After taking informed consent, we prospectively enrolled children with SAM into our study lasting the first 3 days of admission. For the StoolSAM study we assessed stool frequency and consistency prospectively in 120 SAM patients (see below). We compared the current MOYO practice of maternal-reported stool frequency (with a picture aid to help accurate recall during the previous night/day, see Fig. 1) versus a ‘gold standard’ in which a health care worker observed stool frequency using diapers.

As ‘gold standard’ (the direct observation method) diapers were assessed for presence and consistency of stools every 2 h during office hours (8 AM-6 PM), 7 days a week, by one of two members of the study team (AB, IP), thereafter we noted the number of diaper-changes made by the caregiver.

For the maternal recall, we used the standard departmental questionnaire administered during the clinicians’ morning ward round, roughly between 9 and 11 am each day. Supporting the questionnaire, primary care givers were shown a picture aid to help accurate recall of stool frequency during the previous 24 h night and day period (see Fig. 1). For simplicity, we here use the common term ‘maternal recall’ as primary care givers were by and large the actual mother of the child. Study patients were assessed both on week as well as weekend days. We did not have the resources to directly and regularly observe diaper-based output throughout the night (6 PM-8 AM). However, primary care givers were allowed to change diapers as needed, enabling the study team to count the diapers used the following morning and tally the total diapers used in each 24 h period. Diapers were observed by the study team to confirm watery stool rather than urine.

After completing the 3-day study, guardians were asked which of the two methods they preferred and what level of education they had obtained. To assess maternal preference, we used a 1–5 scale: 1: strongly prefers diapers, 2: prefers diapers a little, 3: doesn’t mind which method was used, 4: prefers recall method a little, 5: strongly prefers recall method. Guardians were then prompted to explain their choice (free text) on the above-mentioned 5-point scale.

We initially designed, registered and conducted the study as an RCT (see Additional file 1 for study flow chart). First, we aimed to establish whether stool frequency reported by care givers differed if diapers were also being directly assessed by staff health workers; insuring that “maternal reporting” is representative of normal practice even though direct observation is also being conducted. This also allowed us to check if diarrhoea prevalence estimated with maternal recall was the same in group-1 (“recall only” – unbiased by seeing diapers) and group-2 (“recall *and* diaper observation”). Recognising the limitations of this design (i.e. the assumption that true numbers of diarrhea stools are the same in the two groups) we also performed a post-hoc secondary analysis focusing on the discrepancies in stool frequency obtained by the two methods in each individual child of group-2.

The randomization sequence was computer generated. Allocation concealment was achieved by inserting group labels into sealed, sequentially numbered opaque envelopes. At enrolment, the guardian drew the next numbered envelope and opened it in presence of a study team member to show their assigned group.

### Analyses and statistics

#### Group-1 (baseline – maternal recall only) compared to group-2 (maternal recall with introduced diaper observation)

Difference in stool frequency by maternal recall between group-1 and group-2 were assessed using generalized linear models with a Poisson distribution error for count data. Difference between the 2 groups in diarrhoea prevalence as obtained by maternal recall was assessed with Fisher Exact test.

#### Group-2 analysis – comparing maternal recall to direct diaper observation

We took our ‘gold standard’ measurement of diarrhoea as having three or more diapers with ‘loose’ or worse consistency during the 14-h daytime observation period. The sensitivity and specificity of using stool frequency assessed by maternal recall to classify children as having diarrhoea or not were estimated with 95% confidence intervals for both day 1 and day 2. We analyzed all patients with sufficient data to be classified. Since maternal recall is always retrospective in nature and diapers prospective, day 2 recall was compared with day 1 diaper data, and similarly, day 3 recall was compared with day 2 diaper data.

We recruited 58 children for group-2 (with both recall and diaper observation): this sample size assumed that a clinically relevant limit of agreement would be ±1.5 stool episodes per day (alpha = 0.025; beta, power = 80%; SD 2.5). These figures were based on a review of recent case notes of patients admitted to our ward. To establish agreement, mean differences and limits of agreement between methods were calculated with the R package MethComp [11] which is based on the Bland-Altman approach [12]. Generalized linear models with Poisson error distribution were used to relate stool frequency and primary care giver education with absolute discrepancy between methods. For analysis, WHZ, WAZ, HAZ and MUAC Z-scores were calculated using the WHO Child Growth Standards R package: igrowup [13]. Data entry was done with Microsoft access. Stata version 12.0 (StataCorp USA), SPSS and R (Version 3.2.3) were used for further analyses. Significance threshold was set at 5% for all statistical tests.

### Ethics

The Malawi College of Medicine Research and Ethics Committee approved this study (P.07/13/1429) and all research associated activities were carried out according to Good Clinical Practice guidelines which are based on the Declaration of Helsinki [14].

## Results

Between November 2013 and February 2014, 307 children were admitted to ‘Moyo’ Nutritional Rehabilitation and Research Unit (NRU) and 120 children with SAM were enrolled in the “StoolSAM study”, 113 had data for recall analysis; 55 in *group-1 (baseline – maternal recall only)* and 58 in *group-2 (maternal recall with direct diaper observation)*. Of these patients in group 2, 52 had both recall and diaper observation data for day-1 and 50 for day-2. The children had a median age of 20.5 months and had almost 2 weeks of co-morbidity symptoms (13.5 days) before admission to Moyo; also, all study children were reported to have had a median of 3 days of consistent diarrhoea prior to admission (Table 1). Most primary care givers had only attended primary school (70.2%) or had not received any formal education (3.5%). Kwashiorkor prevalence was high in this cohort (56.9%). The majority, 64% (37/58) of patients, presented with diarrhoea and 33% (19/58) were HIV sero-positive. Most children, 81% (47/58) completed 3 days of stool frequency recording, 3 patients died during recall assessment and 1 patient absconded. The overall mortality rate during the ‘StoolSAM’ trial was 16% (9/58).

**Table 1:** Patient characteristics of Group-2 at baseline

Patients characteristics (*n* = 58)	N (%) *or* Median (Q_1_; Q_3_)
Female	29 (50.0%)
Age of child (months)	20.5 (12.0; 26.0)
Age of primary carer (years)	30 (23; 32)
Education of primary carer:	
None	2 (3.5%)
Primary school	40 (70.2%)
Secondary school	14 (24.6%)
University	1 (1.8%)
Diarrhoea present on admission	37 (64%)
Days of diarrhoea prior to admission	3 (0; 8)
HIV reactive	19 (33%)
Weight for Height Z-score (WHZ)	-3.1 (−3.9; −2.1)
Weight for Age Z-score (WAZ)	-3.6 (−4.7; −2.8)
Length for Age Z-score (LAZ)	-2.8 (−4.3; −2.1)
MUAC for age Z-score	−3.0 (−3.8; −2.2)
Non-oedematous malnutrition:	
by WHZ < −3 or MUAC < 11,5 cm	23 (39.7%)
by WHZ only (WHZ < −3 and MUAC > =11,5 cm)	7 (12.1%)
by MUAC only (WHZ > = − 3 and MUAC < 11,5 cm)	4 (6.9%)
Oedematous malnutrition:	33(56.9%)
Oedema: +	8 (13.8%)
Oedema: ++	14 (24.1%)
Oedema: +++	11 (19.0%)

No differences were found in stool frequency reported by maternal recall between group-1 *(baseline – maternal recall only)* and group-2 *(maternal recall with introduced diaper observation)* (On all 3 days, the differences between the 2 groups in reported stool-frequencies were between 0.9 and 1.07 stools/day, 95% Confidence Intervals between: 0.65–1.38, and *P* values between: 0.25–0.62, > 0.05 showing no difference, see Additional file 1). Furthermore, no difference in diarrhoea prevalence as calculated by maternal recall on day 1 or day 2 were found between the two groups (both day-1 and day-2; *p*-value =1).

The analysis of group-2 showed poor agreement between maternal recall and the ‘gold standard’ of directly observed diapers by health care workers. The prevalence of diarrhea and diagnostic accuracy of next day maternal recall compared with the diaper-observed method as ‘gold standard’ are summarized Table 2. Overall sensitivity was poor, with only 75 and 56% of cases of diarrhoea detected using the maternal recall data on days 1 and 2, respectively. The specificity was higher with 80% or more of children with no diarrhoea correctly identified each day. Positive predictive values (PPV, (95% CI) ranged from 88% (65–97%) to 74% (54–87%) on days 1 and 2, respectively. Negative Predictive Values (NPV, (95% CI) ranged from 65% (53–75%) to 86% (74–93%) on days 1 and 2, respectively (see Table 2 for Prevalence of diarrhoea, PPV and NPV). On day 1, the mean stool frequency difference between the two methods was −0.17 (SD; 1.68) with calculated 2.5 and 97.5% limits of agreement of −3.55 and 3.20 stool per day and, similarly on day 2, the mean difference was −0.2 (SD; 1.59) with limits of agreement of −3.38 and 2.98 stools per day. These calculated limits are well beyond the pre-specified ‘acceptable’ limits of agreement established at ±1.5 stool per day; and this confirms non-agreement of the 2 methods. The average mean difference in stool frequency between the methods (−0.19) does not support a significant systematic bias towards under- reporting stool frequency by primary care givers.

**Table 2:** Prevalence of diarrhoea and diagnostic accuracy of next-day maternal recall compared with diaper observation of stool as the ‘gold standard’ method

Day	Diarrhoea Prevalence ‘Gold Standard’^a^	Specificity (95% CI)	Sensitivity (95% CI)	PPV (95% CI)	NPV (95% CI)
1	20/52 (38%)	94% (80 to 99%)	75% (51 to 91%)	88% (65 to 97%)	86% (74 to 93%)
2	25/50 (50%)	80% (61 to 94%)	56% (35 to 76%)	74% (54 to 87%)	65% (53 to 75%)

^a^Data presented are those of children that had completed both diaper observations and maternal recall on each given day

*PPV* positive predictive value, *NPV* negative predictive value

**Fig. 2:**
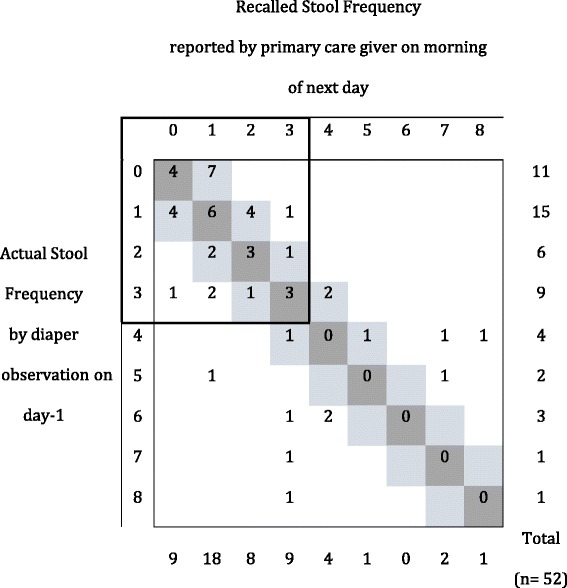
Cross-tabulation of actual diaper observed and recalled stool frequencies for day 1. *Dark grey* cells indicate concordance between stool frequency by recall and diaper observation; *light grey* cells indicate acceptable levels of difference (i.e. not greater than limit of agreement of ±1.5 stool counts per day)

**Fig. 3:**
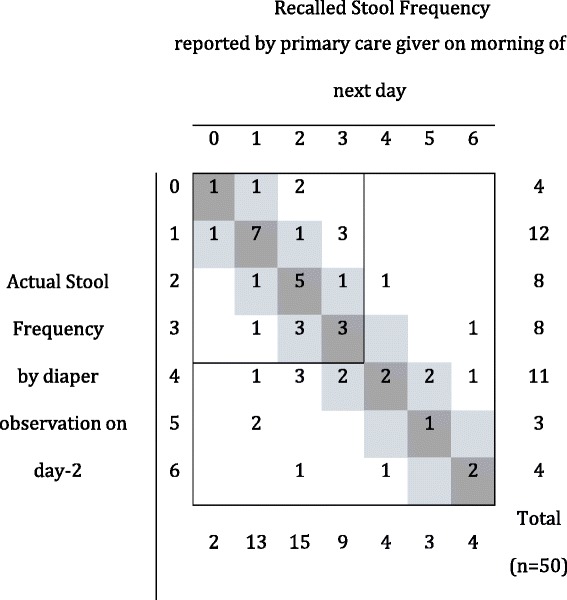
Cross-tabulation of actual diaper observed and recalled stool frequencies for day 2. *Dark grey* cells indicate concordance between stool frequency by recall and diaper observation; *light grey* cells indicate acceptable levels of difference (i.e. not greater than limit of agreement of ±1.5 stool counts per day)

However, it was clear that the higher the stool frequency, the more discrepant the two methods were (see Figs. 2 and 3); where a strong association was found between increased stool frequency and absolute difference between methods (day 1: 1.33 (95%CI: 1.20 to 1.48; *p*-value <0.001); day 2: 1.20 (95%CI; 1.03 to 1.41; *p*-value = 0.017).

The education level of the primary care giver may also influence the deviation between maternal recall and direct diaper observation. The absolute difference between maternal recall and direct diaper observation was significantly associated with years of education on day 1 (0.93; 95% CI 0.87 to 0.99; *p*-value = 0.03); with higher educated mothers reporting stool frequency counts that showed less discrepancy with direct diaper observation. However, this relationship is unclear as it was not found to be significant on day 2 (0.97; 95% CI 0.90 to 1.03; *p*-value = 0.3).

Preference between the two methods was obtained for 47 primary care giver/child pairs. Most primary care givers strongly preferred diapers: 95.7% (45/47). The main reasons given were: the accuracy of evaluating the child’s illness (21/47); better hygienic conditions for the child, i.e. bedding and clothes are not soiled, (15/47); less time on laundry and more with the child (8/45).

## Discussion

To identify those children with increased risk for mortality it is important to have accurate indicators of diarrhoea regarding both frequency and consistency. Our results clearly show there is non-agreement between maternal recall and a ‘gold standard’ of directly observing stool frequency using diapers (see Methods) in children with *complicated* SAM. The higher the stool frequency was, the more the two methods differed suggesting that maternal recall becomes increasingly inaccurate when stool frequency becomes most critical clinically. Although practitioners anecdotally report that primary care givers tend to under-report the stool frequency of their child, there was no evidence to support a systematic negative bias in our study. The educational level of the primary care giver may improve accuracy of stool frequency recall; but this tendency was inconsistent and should be re-investigated.

Diarrhoea is notoriously difficult to assess. It is, however, a highly important clinical measurement as it relates strongly to clinical outcome. Being confident that your measurement of stool frequency and consistency is accurate and precise is therefore essential. In the latest WHO update on the management of SAM [15] several research priorities were detailed including assessment and management of diarrhoea. Although not designed to detect improved care, the results from this small trial suggest that implementing this new method could be both useful for improving patient care and for enhancing the informative quality of diarrhoea outcome in future clinical studies.

There are several limitations in this study. First of all, with 113 enrolled patients and 58 available for method comparison, our sample size is small. Second, we were unable to directly observe diapers 24 h per day; and doing so for 10 h per day was the nearest we could get to the ‘gold standard’. The design of the trial might have biased the outcomes; having nappies could have influenced recall and running a study on stool frequency could have heightened the general awareness to diarrhoea; the ‘Hawthorne effect’ [16]. This could have influenced the recall performance of our baseline group-1. In addition, using our picture aid (Fig. 1) could also have favourably influenced maternal recall. This entails that in ‘normal’ practice, the anecdotal reports by physicians of under-reporting a sick child’s stool frequency may still be a real cause for concern. Conversely, there is no one definite “gold standard” method (other than continuous observation by a health professional). The diaper method may also underestimate the true number of abnormally loose/watery stools. Considering that the established clinical cut off to categorize a child as having diarrhoea or not is 3 or more loose/watery stools per day, the margin for error is small.

We did not look whether the choice of method would influence clinical practice or if it would change the number of days of admission to the ward. Although collecting diapers 2-hourly is a huge demand on personnel, when stool frequency is the primary outcome in a SAM research project, we argue that the direct observation of diapers should be used. Therefore, the method described in this paper might contribute to better outcomes by accurately estimating frequency of diarrhoeal stools (in an in-patient setting with low resources).

In many ways, Moyo NRU is a typical ward in a poor resource setting, but in light of the research conducted in this NRU, its resources are superior to many other NRU’s across the country and elsewhere in Sub-Saharan Africa. Because Moyo NRU is a relatively well-equipped NRU, questions could arise over generalizability of the results. Nevertheless, it is likely that recording of defecation frequency in any NRU, in less well-supported settings, is highly unreliable. We call for other nutritional rehabilitation units in middle- and low-income countries to also investigate this issue as the benefits in doing so could be significant.

High-income countries do rely on stool frequency recalled by parents (19) but the usage of diapers is ubiquitous. In the high-come setting, the cost of diapers is not a concern when designing trials. In Malawi, the cheapest diapers cost 200 MWK (0,45 USD). The mean number of diapers used in this study was 15 so per child this adds to less than 2 USD per day of study. This is above the daily budget of many families of children admitted with complicated SAM. Another issue with regards to using disposable diapers is the waste associated with it and the energy costs to make diapers.

## Conclusions

In conclusion this study shows non-agreement between the assessment of stool frequency in admitted SAM patients using maternal recall and direct observation of diapers by health care staff. It shows that relying only on maternal recall is inaccurate in measuring the incidence/prevalence of diarrhoea, as the reported number of loose/watery stools may not reflect the actual number. The use of diapers to record stool frequency in SAM patients might be a promising method but more studies in other low-income settings are needed before recommending more widespread use. Clinically, we recognize a different cost/benefit balance than in resource rich settings especially given that SAM is only really common in resource poor settings. We suggest to selectively use diapers for high-risk children (e.g. for those with complicated SAM, admitted to a NRU), where stool frequency monitoring is crucial. The results of this trial should influence the design of future trials in malnutrition research; especially where stool frequency is the primary outcome.

## Additional files

Additional file 1: Frequency distribution of recalled number of stools, by RCT study arm: group-1 *(baseline – maternal recall only, n = 55)* and group-2 *(maternal recall with direct diaper observation, n = 58).* No evidence was found to suggest that stool frequency as obtained by maternal recall in group-1 and group-2 differed on any day of the study as tested by generalized linear models with Poisson error distribution for count data. (DOCX 84 kb)

Additional file 2: StoolSAMdata_Submission, .csv file, contains all data underlying this paper in an anonymised manner. (CSV 41 kb)

## Abbreviations

HAZ: Height for age z-score
HIV: Human immunodeficiency virus
MUAC: Mid upper arm circumference
MWK: Malawian Kwacha
NPV: Negative predictive value
NRU: Nutritional rehabilitation unit
PPV: Positive predictive value
QECH: Queen Elizabeth Central Hospital
SAM: Severe acute malnutrition
USD: United states dollar
WAZ: Weight for age z-score
WHZ: Weight for height z-score
