# Supplementary figures and images for: Stool frequency recording in severe acute malnutrition (‘StoolSAM’); an agreement study comparing maternal recall versus direct observation using diapers
Source: BMC Pediatr. 2017 Jun 7;17:140. doi: 10.1186/s12887-017-0874-0 (PMC5461774; doi:10.1186/s12887-017-0874-0)

**
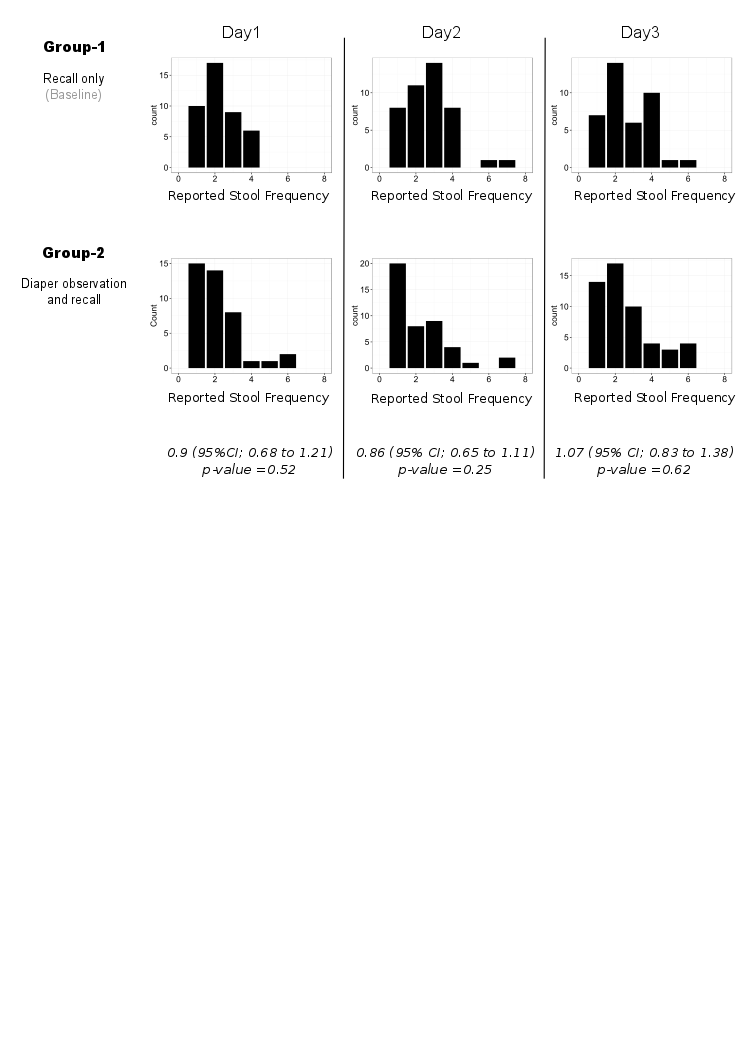
**

Supplement: Supplementary file 1 — Frequency distribution of recalled number of stools, by RCT study arm: group-1 (baseline – maternal recall only, n = 55) and group-2 (maternal recall with direct diaper observation, n = 58). No evidence was found to suggest that stool frequency as obtained by maternal recall in group-1 and group-2 differed on any day of the study as tested by generalized linear models with Poisson error distribution for count data. (DOCX 84 kb) [file 12887_2017_874_MOESM1_ESM.docx]
